# Introducing the carbon footprint reduction index (CaFRI) as a software-supported tool for greener laboratories in chemical analysis

**DOI:** 10.1186/s13065-025-01486-2

**Published:** 2025-05-09

**Authors:** Fotouh R. Mansour, Paweł Mateusz Nowak

**Affiliations:** 1 Department of Pharmaceutical Analytical Chemistry, Faculty of Pharmacy, Tanta University, Tanta, 31111 Egypt; 2 Medicinal Chemistry Department, King Salman International University (KSIU), Ras Sudr, 46612 Egypt; 3 Department of Analytical Chemistry, Faculty of Chemistry, Jagiellonian University in Kraków, Gronostajowa St. 2, Kraków, 30-387 Poland

**Keywords:** Carbon footprint, CaFRI, Sustainability, Analytical chemistry, Environmental impact, Waste management, Energy efficiency, Greenhouse gas emissions

## Abstract

Carbon Footprint Reduction Index (CaFRI) has been presented as a newly developed web tool designed to assess and enhance the sustainability of analytical methods, with a focus on estimating greenhouse gas emissions (available at bit.ly/CaFRI). While many tools exist for evaluating greenness, none specifically address the carbon footprint of laboratory procedures. CaFRI fills this gap by providing a standardized approach that predicts the effectiveness of carbon footprint reduction strategies. It assigns a numerical rating based on direct CO_2_ emission factors such as energy efficiency and indirect factors like sample storage, transportation, waste management, and reagent use. By implementing CaFRI, laboratories can optimize resource use, minimize environmental hazards, ensure compliance with eco-friendly regulations, and target specific areas for improvement. Case studies using techniques such as spectrophotometry for polidocanol in ampoules, dispersive solid phase microextraction with HPLC/UV for ritonavir in human plasma, carbon quantum dots for molnupiravir in capsules, and homogenous liquid-liquid microextraction with HPLC/UV for favipiravir in human plasma demonstrated CaFRI’s applicability in evaluating the carbon footprint across diverse analytical methods and matrices. These case studies illustrated that energy consumption and CO_2_ emissions are key criteria for CaFRI scores, with higher scores indicating more sustainable methods.

**Supplementary Information:**

The online version contains supplementary material available at 10.1186/s13065-025-01486-2.

## Introduction

The carbon footprint is a quantitative assessment of the combined emissions of greenhouse gases, including carbon dioxide (CO_2_), methane (CH_4_), and nitrous oxide (N_2_O) in terms of their carbon dioxide equivalent (CO_2_e) [1]. These emissions are produced as a result of human activities, both directly and indirectly. It measures the extent to which these activities affect the environment by assessing their role in causing global warming and climate change. Carbon footprints primarily contribute to climate change by causing the retention of heat in the Earth’s atmosphere [2]. This leads to an increase in global temperatures, more frequent occurrences of extreme weather events, and disturbance of ecosystems. Elevated levels of carbon footprints indicate also the risk of emission of pollutants into the atmosphere, the acidification of oceans, and the depletion of forests, resulting in the destruction of habitats, the decline of biodiversity, and detrimental impacts on ecosystems [3]. Moreover, elevated levels of greenhouse gas emissions resulting from high carbon footprints can give rise to respiratory ailments, cardiovascular disorders, and other health complications as a consequence of air pollution and inadequate air quality. For these reasons, it is crucial to reduce the environmental impacts and undertake transition towards a more sustainable future for both ecosystems and human societies. This can be achieved by reducing carbon footprints through sustainable practices, energy efficiency, renewable energy adoption, waste reduction, and conservation activities.

Metrics are essential for evaluating the decrease of carbon footprint in analytical chemistry laboratories [4, 5]. They offer a systematic method for measuring and improving energy usage, waste creation, and greenhouse gas emissions. By monitoring metrics, laboratories may pinpoint areas that need improvement, enhance the efficient use of resources, and lower operational hazards. These goals can be achieved, while ensuring compliance with environmental rules and sustainability requirements. Metrics also enable the assessment of analytical tools and methodologies, promoting the advancement of more environmentally friendly practices. Moreover, comparing performance against established industry standards and exemplary practices enables laboratories to improve their environmental performance, increase awareness, and contribute to a more sustainable future. Accordingly, the strategic utilization of metrics to evaluate the reduction of carbon footprint in analytical chemistry laboratories not only improves operational efficiency and adherence to regulations, but also establishes sustainable practices within the scientific community and beyond.

Despite several greenness metrics dedicated to analytical methods are available and willingly used by analytical chemistry community, e.g. AGREE [6], GAPI [7], MoGAPI [8], ComplexMoGAPI [9], Analytical Eco-Scale [10], RGB [5], ChlorTox Scale [11], etc., it lacks the one focused primarily on carbon footprint. While criteria such as energy consumption appear in some models, they are typically not the most important, and the general purpose of known metrics is to capture overall greenness or risks related to chemical exposure, not greenhouse effect. Therefore, there is a significant need for the development of a new tool allowing to reduce emissions in analytical chemistry laboratories [12, 13]. Reduction of greenhouse gas emission is a crucial component of greenness [14] but somewhat overlooked and remaining in the shadow of threats directly related to the chemical impact of the reagents and solvents used.

A few initiatives and tools have emerged to assess and monitor carbon footprints, such as GES 1.5 [15] and the UK Carbon Trust [16]. GES 1.5 is an open-source web application designed to estimate and reduce the carbon footprint of research labs, departments, and teams by analyzing emission sources like buildings, commuting, digital devices, and professional travel. Meanwhile, the Carbon Trust operates on a broader scale, working with businesses and public organizations to support carbon reduction initiatives and foster innovation for a low-carbon economy. While both tools contribute significantly to sustainability efforts, they function at an institutional or organizational level rather than addressing emissions at the granular level of specific analytical procedures.

This study presents the Carbon Footprint Reduction Index (CaFRI) as an innovative tool for evaluating analytical procedures and the efficiency of carbon footprint reduction initiatives adopted in chemical laboratories. This tool represents a notable advancement in promoting environmental sustainability principles in laboratory settings. The Intergovernmental Panel on Climate Change (IPCC) provides internationally recognized methodologies for calculating greenhouse gas emissions, primarily based on the quantification of carbon dioxide equivalents (CO_2_e) using global warming potential (GWP) values for various substances.

## Carbon footprint reduction index (CaFRI)

**Fig. 1:**
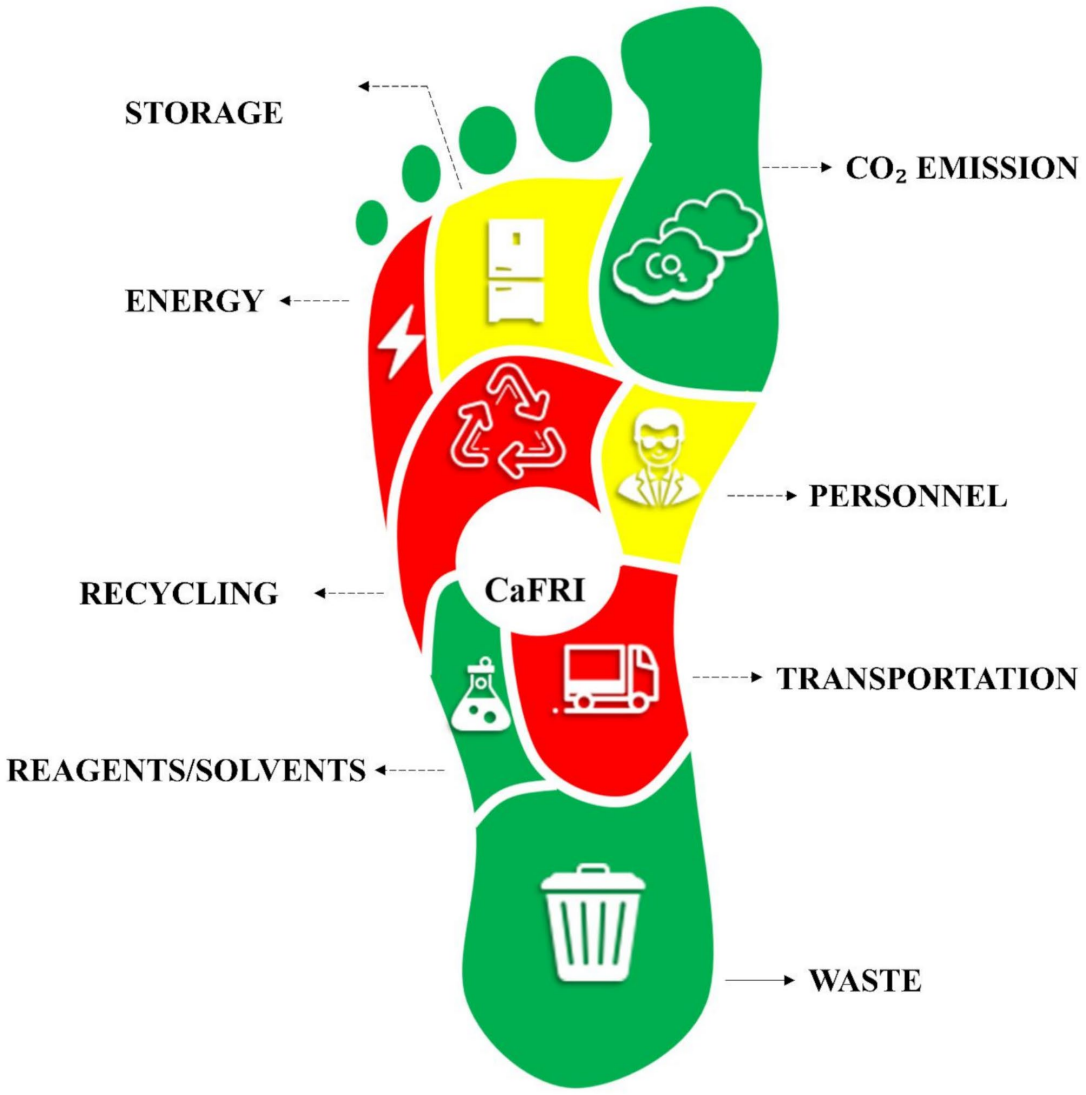
The pictograms of the proposed CaFRI assessment, and the various parameters contributing to the total score

The CaFRI is a comprehensive greenness assessment tool prioritizing carbon footprint as the primary environmental impact, dedicated to already developed analytical laboratory procedures. It takes into account the specification of analytical method as well as circumstances dependent on the laboratory where the method was utilized. The evaluation takes into account several important factors, including energy demand, emissivity of energy production, the application of specific carbon footprint reduction measures, sample storage, transportation, personnel, waste management, recycling efforts to minimize resource usage, and use of chemicals. The assessment of energy efficiency is conducted in a simple way, by estimating the total electric power of electric devices and sample throughput. This and other criteria are outlined in Table 1. The components of this metric are elaborated upon in depth in the subsequent sections. Figure 1 illustrates the various parameters included in the CaFRI assessment and how they contribute to the overall score. The maximum possible score of any analytical method according to the CaFRI assessment is 100.

**Table 1:** Parameters used to calculate the total score in CaFRI

Parameter	Choices	Points
**ENERGY**		
An energy reduction program or clean energy sources are adapted throughout the procedures	Yes	4
	No	1
Total electrical power use of analytical instruments	< 0.1 kW	5
	0.1–1.5 kW	3
	> 1.5 kW	1
Energy-intensive non-analytical equipment is essential (fume hood, air conditioners)	Yes	1
	No	4
Number of samples analyzed per hour	> 30 samples/h	3
	10–30 samples/h	2
	< 10 samples/h	1
**CO**_**2**_ **EMISSION**		
The carbon footprint of the electrical power of analytical instruments is known	Yes	4
	No	1
Emission factor	< 0.1 kg CO_2_/kWh	5
	0.1–0.3 kg CO_2_/kWh	3
	> 0.3 kg CO_2_/kWh	1
**STORAGE**		
Sample Storage	No storage was required	3
	Storage under normal conditions (refrigerators)	2
	Storage under special conditions (deep freezers, vacuum, high pressure)	1
**TRANSPORTATION**		
The sample has to be transported to an analytical Laboratory	No	2
	Yes	1
Distance between the sample field and the laboratory	< 1 mile	3
	1–10 miles	2
	> 10 miles	1
Number of samples transported in one shipment	> 100 samples per shipment	4
	11–100 samples per shipment	3
	2–10 samples per shipment	2
	1 sample per shipment	1
An ecofriendly vehicle is used in transportation	Yes	2
	No	1
**PERSONNEL**		
Number of personnel required for one sample analysis	1 person	4
	2–3 persons	3
	4–5 persons	2
	> 5 persons	1
Automation	automatic	3
	semiautomatic	2
	manual	1
**WASTE**		
Waste amount	< 10 mL or g per sample	3
	10–100 mL or g per sample	2
	> 100 mL or g per sample	1
Waste disposal	Waste disposal by a specialized personnel/entity	3
	Waste disposal by the analyst	2
	No waste disposal is performed	1
**RECYCLING**		
	The method employs recycled reagents/solvents from the same method	3
	The method employs recycled reagents/solvents from other methods	2
	No recycling is performed	1
**REAGENTS/SOLVENTS**		
Total number of pictograms	≤ 3	4
	4–6	3
	7–9	2
	> 9	1
Total amount of organic solvents per sample	< 5 mL	3
	5–10 mL	2
	> 10 mL	1
Total amount of reagents per sample	< 1 g or mL	3
	1–3 g or mL	2
	> 3 g or mL	1

The tool is integrated with the user-friendly software, freely available on the website (https://bit.ly/CaFRI*).* After selecting the appropriate answers in questionnaire, the result of assessment is presented in the form of a pictogram in the shape of a human foot, which is directly associated with the carbon footprint. Different places on the foot represent the corresponding criteria. Red color corresponds to a poor rating, yellow to an average rating, and green to a good rating, in line with the idea of ​​green chemistry. The number of points obtained from the questionnaire is converted into a final result on a scale of 0-100. The ideal procedure (fully green) in terms of carbon footprint estimation gains a score of 100. Points are assigned based on the significance of each parameter in relation to the carbon footprint. This explains e.g. why the emission factor is allocated more points compared to sample storage or transportation.

### Energy consumption

The assessment of energy usage is crucial in determining a laboratory’s environmental impact within the CaFRI [17]. The first criterion takes into account the implementation of a dedicated energy production/utilization program during procedures, e.g. the employment of local green energy sources, such as solar cells or air turbines, dedicated to powering the specific laboratory – which relates to energy production, or utilization such as regular energy audits and use of unconventional energy-efficient equipment, which relates to energy conservation. The higher ratings are assigned to laboratories that employ such measures.

As the second criterion, the energy consumption per sample is considered. It is done in two steps. In the first one the overall energy demand of research instruments is estimated by adding up their electric powers (kW). Obviously, better ratings are given when instruments consume less energy. Therefore, the CaFRI encourages laboratories to adopt energy-saving practices and utilize energy-efficient equipment. This can trigger efforts to reduce their carbon footprint and strive for more sustainable operations. In the second step sample throughput is considered, since the total energy demand per sample depends both on instruments and on how long they are used. The detailed calculation considering independently each device and its operation time could be problematic, therefore, to maintain user-friendliness, the assessment is done in the simplified way. To facilitate estimation of electric power, Table 2 lists the various popular analytical equipment. These data are based on self-assessment by two independent chemical engineers. It should also be noted that if the energy consumption of the given instrument could not be accurately estimated or verified experimentally (using e.g. wattmeter), the maximum possible value of electric power should be considered in the calculations.

**Table 2:** Approximate electrical power for various analytical techniques in kilowatts (kW)

Instrument	Expected power (kW)
HPLC (High-Performance Liquid Chromatography)	0.5–1.5
UHPLC (Ultra-High-Performance Liquid Chromatography)	0.8–2.0
LC-MS (Liquid Chromatography-Mass Spectrometry)	1.0–3.0
GC (Gas Chromatography)	1.0–2.5
GC/MS (Gas Chromatography-Mass Spectrometry)	1.5–3.5
UV/Vis (Ultraviolet-Visible) Spectrophotometer	0.1–0.3
Spectrofluorometer	0.2–0.5
Potentiometer	0.05–0.1
Voltamograph	0.05–0.1
Polarograph	0.05–0.1
Capillary Electrophoresis	0.2–1.5
ICP/MS (Inductively Coupled Plasma Mass Spectrometry)	2.0–5.0
AAS (Atomic Absorption Spectroscopy)	0.5–1.5
FTIR (Fourier Transform Infrared Spectroscopy)	0.1–0.3
Raman Spectrometer	0.1–0.4
NMR (Nuclear Magnetic Resonance)	1.5–4.0
TOC Analyzer (Total Organic Carbon)	0.1–0.5

Additionally, the energy intensive non-analytical equipment such as fuming hoods and air conditioners are considered to ensure that the proposed energy estimation scheme is comprehensive. If the analytical procedure does not require them, a higher point number is awarded.

### CO_2_ emissions

**Fig. 2:**
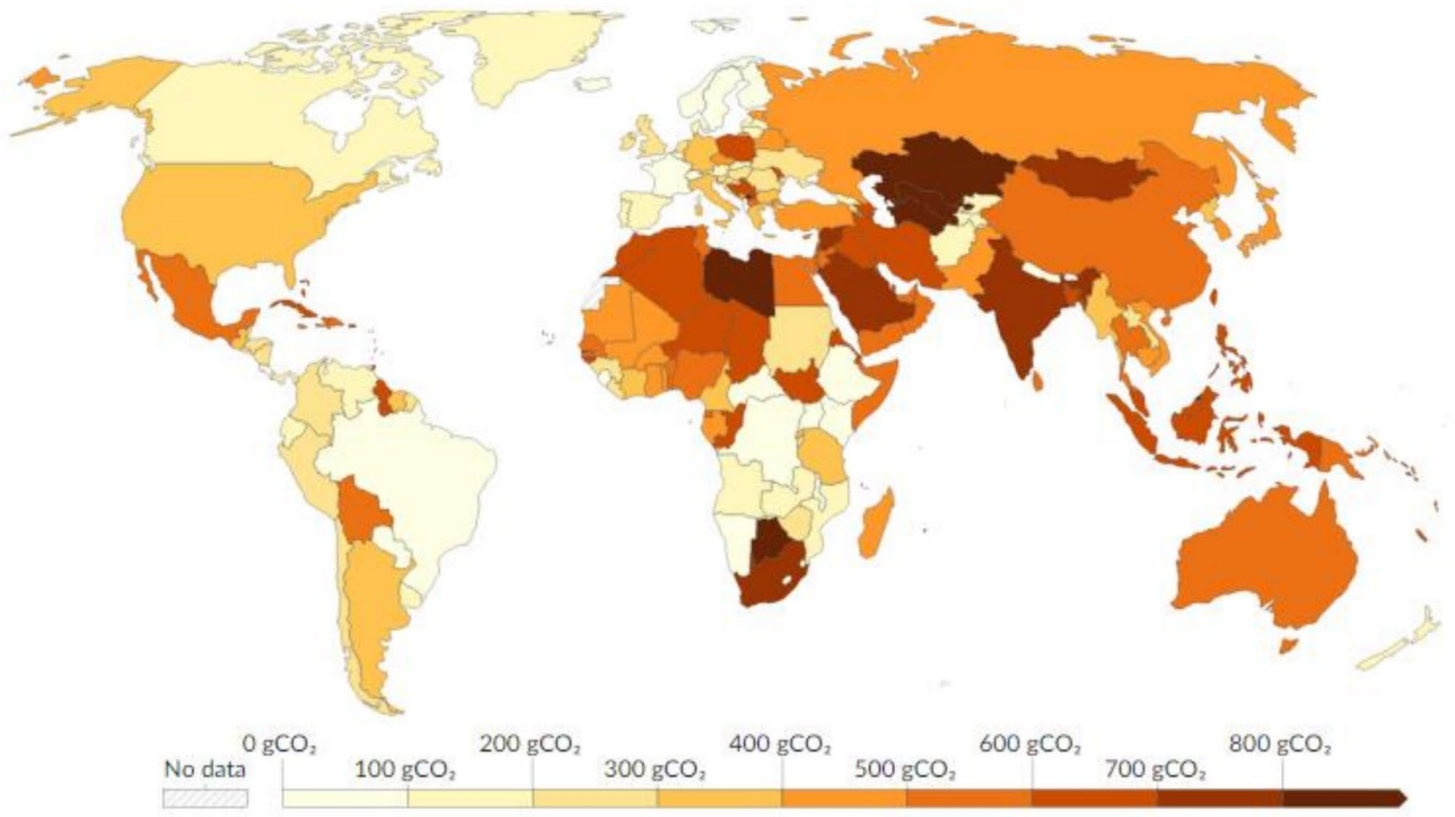
Country-specific carbon intensity, expressed in g of CO_2_-equivalents emitted per kWh of electricity generated in 2023 (With permission from [19])

Admittedly, accurately evaluating the amount of CO_2_ emissions is crucial for comprehending and minimizing the environmental consequences of laboratory activities [18]. Therefore, the CaFRI promotes laboratories which as a rule of thumb, proactively measure and oversee their emissions (using e.g. life-cycle assessment procedures), which can result both from using given analytical procedures and maintaining working infrastructure, e.g. ventilation, lighting, room heating. In addition, this section takes into account the emission factor related to energy production, assigning higher marks to lower emission factors per kilowatt-hour (kWh) of energy. This parameter is geographically specific, it can vary from less than 50 g of CO_2_ (Sweden, Norway) to over 0.8 kg (Libya, Kazakhstan), depending on the share of renewable energy sources in energy production profile [19]. CaFRI users are prompted to account for all known direct and indirect sources of carbon emissions relevant to the analytical process, including high-GWP gases (e.g., SF_6_) when applicable. The tool allows for flexibility and adaptability depending on the laboratory’s specific materials and practices. If measuring CO_2_ emissions directly is not feasible, the updated data available on the Our World in Data website can be utilized to estimate carbon intensity for specific geographical locations [19]. This dataset, spanning from 1990 to 2023, provides information on greenhouse gas emissions per kilowatt-hour of electricity produced for various countries and regions. It serves as a valuable resource for assessing emission levels and tracking the progress of transitioning to more sustainable energy sources. Figure 2 shows the country-specific carbon intensity, expressed in g of CO_2_-equivalents emitted per kWh of electricity generated in 2023.

### Sample storage

When evaluating carbon footprint of analytical procedure, it is crucial to consider how samples and reagents are stored [10]. The grading method assesses the necessity of sample storage, assigning more points to procedures and laboratories that do not require any storage facilities, therefore reducing overall energy usage. In addition, the assessment scheme differentiates between storage under typical and exceptional conditions, assigning low points if the storage in deep freeze (>-60ºC), vacuum, under high pressure or in sterile air is required. This may potentially lead to higher energy consumption and carbon emissions. The CaFRI encourages laboratories to minimize or eliminate the need for sample storage and adopt sustainable storage procedures.

### Transportation

Transportation practices play a critical role in evaluating a laboratory’s efforts to reduce its carbon footprint, according to the evaluation criteria of the CaFRI [20]. The rubrics and grading systems take into account several aspects, including the necessity of transporting samples to an analytical laboratory, the distance between the sample field and the lab, the quantity of samples transported in one shipment, and the utilization of environmentally friendly vehicles. Shorter distances, greater batch sizes in shipments, and the adoption of ecologically friendly transportation techniques result in higher ratings, indicating a laboratory’s dedication to lowering carbon emissions linked to sample transportation. The CaFRI seeks to promote laboratories’ adoption of environmentally friendly transportation methods by offering motives, with the goal of reducing their ecological footprint and supporting broader sustainability initiatives.

### Personnel

The assessment of people factors is also relevant in shaping a laboratory’s efforts to reduce its carbon footprint [21]. Methods that require fewer analysts generally have a smaller carbon footprint since they are simpler, more efficient, and characterized by reduced energy consumption, resource usage, waste production, and transportation emissions. The tool considers the people requirements for a single sample analysis, giving better grades to processes that are efficiently carried out by a less number of individuals. Additionally, the degree of automation in the analysis process is taken into account, with the highest score given to operations that are fully automated. This reflects an efficient and resource-saving method for analyzing samples. The CaFRI aims to promote operational efficiency, energy conservation, and environmental sustainability in laboratories by motivating the optimization of personnel utilization and the integration of automation technologies. This will create a more eco-conscious and sustainable laboratory environment.

### Waste management

Efficient waste management is crucial in evaluating the environmental footprint of a laboratory [22]. Improper disposal of chemical and solid waste can lead to increased greenhouse gas emissions, including CO_2_, from waste decomposition, incineration or biodegradation processes – which could be omitted by employing specialized entities offering eco-friendly waste utilization methodologies. The rating criteria for waste amount assign scores ranging from 1 to 3 based on the quantity of waste generated per sample. Additionally, the rates evaluate waste disposal methods, giving higher scores to disposal by specialized personnel such as environmental health and safety officer, waste management technicians, or professionals from certified waste disposal companies compared to disposal by the analyst or no disposal at all. These rubrics highlight the significance of proper waste handling in efforts to reduce carbon footprint. The CaFRI seeks to encourage laboratories to reduce waste production and implement environmentally responsible disposal methods by providing ratings that reflect their waste generation and disposal procedures. This will ultimately contribute to a more sustainable and environmentally conscious operational strategy.

### Recycling

Assessing recycling methods in a laboratory is crucial for promoting sustainability and reducing carbon footprint, as part of the CaFRI [23]. The lack of recycling necessitates the delivery of newly produced reagents, which creates an additional carbon footprint associated with production and transportation. The rating criteria for recycling assess various levels of environmental awareness, assigning higher grades to approaches that employ recycled reagents and solvents, from the same process. By using recycled materials, laboratories can both decrease resource consumption and lower trash output, resulting in higher scores of the index. The ratings decline for laboratories that employ recycled reagents/solvents from other methods. The lowest score is assigned to facilities where no recycling is performed. The CaFRI encourages laboratories to implement resource conservation methods and contribute to a greener and more sustainable approach to chemical analysis by assigning scores that reflect their recycling efforts.

### Reagents and solvents

As the last criterion, the CaFRI considers the utilization of hazardous reagents and solvents, which influence indirectly the carbon footprint of an analytical procedure, e.g. by the need to use advanced processing technologies at the end of chemicals’ life cycle [24]. The grading method takes into account the total number of hazard pictograms associated with chemical reagents and solvents in safety data sheets, promoting the use of less toxic and hazardous chemicals by awarding more points. This indicates the presence of potentially dangerous or environmentally detrimental materials being used. It can be assumed that more pictograms means that more advanced waste handling methods are needed, which generate more CO_2_ as a side effect. In addition, the assessment considers the overall quantity of organic solvents and reagents used per sample. Labs that use less amounts per sample are given better marks, as this contributes to lesser carbon footprint related to chemical manufacturing process, handling and transport of waste, and its final disposal. The CaFRI encourages laboratories to adopt environmentally-friendly practices by advocating the reduction of hazardous chemicals, decreasing the use of solvents and reagents, and supporting eco-conscious decisions. Such a systemic approach assist laboratories in offsetting their carbon footprint and contribute to achieving environmental sustainability goals.

## Case studies

To demonstrate the applicability of the CaFRI tool, we used it to assess four methods for quantifying different substances: polidocanol in commercial ampoules [25], ritonavir in human plasma [26], molnupiravir in hard gelatin capsules [27], and favipiravir in human plasma [28].

**Fig. 3:**
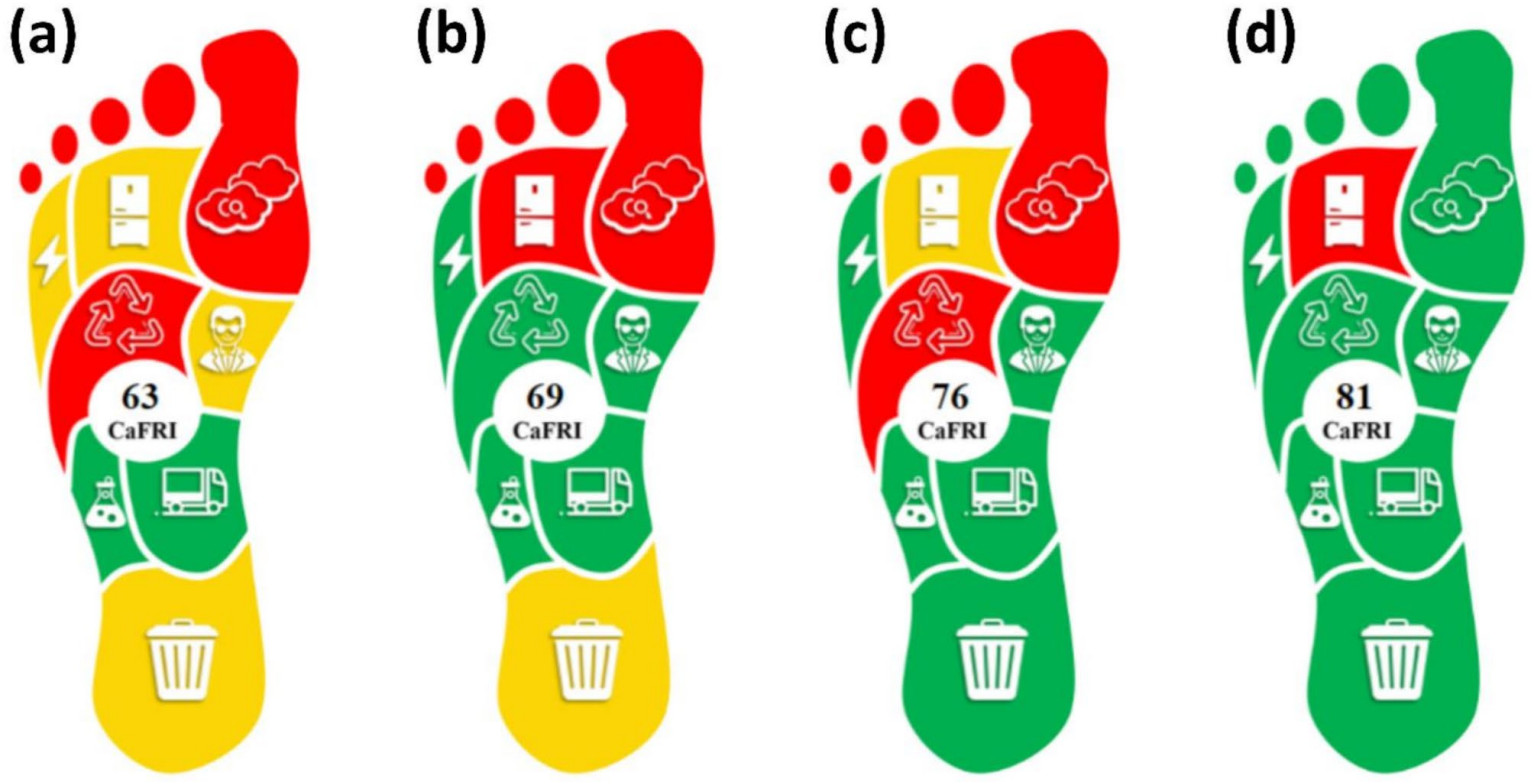
The results of the first (**a**), second (**b**), third (**c**) and fourth case studies (**d**) presenting the application of the Carbon Footprint Reduction Index (CaFRI) for the assessment of four different analytical methods

The first case study utilized spectrophotometry to quantify polidocanol, a difficult-to-measure medicinal compound that lacks chromophoric properties [25]. The assay principle was based on the creation of a ternary complex between polidocanol and a cobalt(II)-thiocyanate complex, which could be transferred into a dichloromethane layer. Researchers precisely quantified polidocanol by measuring its absorbance at 320 nm. The quantity of waste generated was limited, less than 10 mL. However, no waste disposal initiative was reported. The samples were delivered over a distance of less than 1 mile using compressed natural gas vehicles (as an eco-friendly alternative to traditional vehicles), and an energy reduction scheme was implemented for instruments that consumed greater than 1.5 kW. Two individuals were necessary for each manual sample analysis in terms of personnel and the sample throughput was 8 per hour. The carbon footprint was not directly determined. The emission factor was set to exceed 0.3 kg, as suggested per the data on the “Our World in Data” website. The utilization of reagents and solvents was highly effective, using less than 5 mL of organic solvents and less than 1 g of reagents per sample. Additionally, three pictograms represented the solvents and reagents employed and the sample was stored under standard conditions in the refrigerator. The cumulative CaFRI score for this method was 63 (Fig. 3a).

The second case study utilized a hybrid material consisting of microcrystalline cellulose and a metal-organic framework (MOF) to effectively perform dispersive solid phase microextraction for ritonavir from human plasma [26]. The composites functioned as efficient sorbent materials, facilitating the retrieval of ritonavir from human plasma for subsequent analysis. The waste amount was minimal (< 10 mL), but no waste disposal program was reported. The MOF used in sample preparation could be recycled for reuse in the same method. A total number of 10 samples were transported less than 1 a mile using eco-friendly vehicles, and an energy reduction program was applied with instruments consuming more than 1.5 kW. For personnel, 2 individuals were required per sample analysis using an HPLC/UV instrument. The sample throughput was 4 per hour and the carbon footprint was not measured. The emission factor was set to exceed 0.3 kg, as per the reported data. The use of reagents and solvents was efficient, with less than 5 mL of organic solvents and less than 1 g of reagents per sample, and there were three pictograms for the solvents/reagents used. Sample storage was at -80 °C until analysis. Accordingly, the total CaFRI score of this method was 69 (Fig. 3b).

In the third case study, analysts presented a method for the determination of molnupiravir via carbon quantum dots, synthesized from eggshell [27]. The waste amount was < 10 mL, and the waste was disposed by the analyst, although no recycled reagents were employed. A total number of 50 samples were transported less than a mile using eco-friendly vehicles, and an energy reduction program was applied with instruments consuming less than 0.1 kW. Only one analyst was required per sample analysis and the sample throughput was > 30 samples/h. The carbon footprint was not measured. The emission factor was set to exceed 0.3 kg. The use of reagents and solvents was efficient, with less than 5 mL of organic solvents and less than 1 g of reagents per sample, and there were six pictograms for the solvents/reagents used. Sample storage was at normal conditions. Accordingly, the total CaFRI score of this method was 76 (Fig. 3c).

The fourth case study employed menthol as a phase separating agent in homogenous liquid-liquid microextraction of favipiravir from human plasma before being measured by HPLC/UV [28]. The waste amount was < 10 mL and the waste was disposed by a specialized personnel. The menthol used in sample preparation could be recycled for reuse in the same method. A total number of 60 samples were transported less than one mile using eco-friendly vehicles, and an energy reduction program was applied with instruments consuming more than 1.5 kW. For personnel, 1 individual was required per sample analysis by an HPLC with an autosampler and the sample throughput was 12 per hour. The carbon footprint was measured, and the emission factor was found to be 0.30 kg. Less than 5 mL of organic solvents and less than 1 g of reagents per sample were required, and there were five pictograms for the solvents/reagents used. Sample storage was at -80 °C until analysis. Accordingly, the total CaFRI score of this method was 81 (Fig. 3d).

For comparison purposes, the carbon footprint of an alternative method for the determination of the same substance (favipiravir) was assessed [29], yielding a total CaFRI score of 62 (Figure S1). This relatively moderate score may be attributed to the use of the energy-intensive LC-MS/MS technique, which requires specialized personnel for operation. Further efforts could be made to reduce CO_2_ emissions and implement waste recycling to enhance the method’s environmental performance. Performance in the other case can be improved by tracking the red zones in the final pictogram, by considering waste recycling in the first and the third cases, the storage conditions in the second and the fourth case, and the CO_2_ emission parameter in the first three case studies.

The results indicate significant differences in the precautions used to reduce the carbon footprint among the methods. The first two methods demonstrate acceptable measures, with calculated CaFRI scores between 50 and 74. In contrast, the third and fourth methods show appreciable efforts in reducing the carbon footprint, as indicated by scores of 75 or higher. These case studies indicated that the two key criteria for CaFRI scores are energy consumption and CO_2_ emissions. Reducing energy use, especially through clean energy sources and low-energy equipment, plays a crucial role in minimizing environmental impact. Similarly, lowering CO_2_ emissions by using equipment with lower emission factors and measuring the carbon footprint is vital for accurately assessing and reducing the overall carbon footprint of the procedure. Other parameters also contribute to the total score, but to a lesser extent. These parameters not only help assess the analytical procedure but also indicate areas that need further improvement to develop more sustainable methods.

### Comparison with other carbon footprint tracking methods and strategies

The Intergovernmental Panel on Climate Change (IPCC) provides internationally recognized methodologies for calculating greenhouse gas emissions, primarily based on the quantification of carbon dioxide equivalents (CO_2_e) using global warming potential (GWP) values for various substances. However, CaFRI is not intended to quantify total carbon dioxide equivalents (CO_2_e) as IPCC-based models do. Instead, CaFRI is designed to evaluate the efforts made to reduce carbon footprint within analytical methods—offering a practical, awareness-raising tool for chemists during method development. These differing goals and outputs make direct comparisons with IPCC methods inappropriate. Similarly, Labos 1.5 focuses on institutional-scale assessments, while CaFRI offers a granular evaluation at the method level and gives an evaluation of the efforts exerted to reduce carbon footprint, rather than an estimate of tCO_2_e values. Table S1 summarizes the main differences between CaFRI and Labos 1.5 in scope, functional unit, output and applications.

On the other hand, Life Cycle Assessment (LCA) is a comprehensive methodology encompassing all life cycle stages and multiple environmental indicators. By contrast, CaFRI focuses specifically on assessing the carbon footprint of individual analytical methods, with the implicit functional unit being the analysis of one sample by a defined procedure. CaFRI is thus a more targeted, complementary tool—not a replacement for LCA.

## Conclusion

The CaFRI is a robust tool that allows analytical chemistry laboratories to measure, evaluate, and enhance their environmental sustainability with the focus on parameters associated with greenhouse gas emission, which makes it a valuable compliment of the pallet of the existing greenness metrics. It offers a simple and comprehensive method for assessing procedures applied in the specific laboratories rather than non-specific methods without clear utilization circumstances, and thus provide more information allowing to foresee the final environmental consequences. It takes into account various factors including both those directly and indirectly related to CO_2_ emission. The application of the CaFRI may enable laboratories to discover areas for enhancement, optimize resource efficiency, assure regulatory compliance, and showcase their dedication to environmental stewardship. The CaFRI’s capacity to facilitate evidence-based decision making, foster cooperation, and contribute to worldwide endeavors in tackling climate change renders it a vital resource for the analytical chemistry community. The case studies presented in this article demonstrate how the CaFRI may be used to evaluate the carbon footprint of various analytical procedures. These case studies also emphasize the CaFRI’s ability to facilitate positive change and encourage sustainability in laboratory environments. Like any assessment model, CaFRI involves a certain margin of uncertainty and subjectivity. To address uncertainty and enhance reliability, we recommend that at least two independent evaluators assess each method separately. If there were any discrepancies or doubts in the number of points awarded between evaluators, a consensus should be reached through open discussion and the agreed rating should be presented in the final assessment. This approach may help mitigate subjectivity, ensure a more robust and reproducible carbon footprint assessment, and improve the consistency of CaFRI scores across different laboratories. By adopting this metric, analytical chemistry laboratories can take the initiative in spearheading the development of a more sustainable future for the scientific community and beyond.

## Electronic supplementary material

Below is the link to the electronic supplementary material.

Supplementary Material 1

## Data Availability

No datasets were generated or analysed during the current study.

## Declarations

### Availability and requirements

**Project name**: CaFRI.

**Project home page**: https://bit.ly/CaFRI.

**Operating systems**: This project is a web application and can run on any operating system with a browser.

**Programming languages**: HTML, CSS, and JavaScript.

**Other requirements**: A modern web browser (e.g., Chrome, Firefox, or Edge) is required.

**License**: This software is free for academic and non-profit use. Any modifications or derivative works require prior written permission from the author. Commercial use is prohibited without a separate licensing agreement.

**Any restrictions to use by non-academics**: This software is free for academic and research purposes but requires a license for commercial use.
