# Supplementary material for: Introducing the carbon footprint reduction index (CaFRI) as a software-supported tool for greener laboratories in chemical analysis
Source: BMC Chem. 2025 May 9;19(1):121. doi: 10.1186/s13065-025-01486-2 (PMC12065229; doi:10.1186/s13065-025-01486-2)
Supplement: Supplementary file 1 — Supplementary Material 1 [file 13065_2025_1486_MOESM1_ESM.docx]

**Introducing the Carbon Footprint Reduction Index (CaFRI) as a Software-Supported Tool for Greener Laboratories in Chemical Analysis**

*Fotouh R. Mansour^1^*, Paweł Mateusz Nowak^2^*

*^1^ Department of Pharmaceutical Analytical Chemistry, Faculty of Pharmacy, Tanta University, Tanta 31111, Egypt*

*^2^ Department of Analytical Chemistry, Faculty of Chemistry, Jagiellonian University in Kraków, Gronostajowa St. 2, 30-387 Kraków, Poland*

**Supplementary materials**

The work of Challenger et al. [1] on the determination of favipiravir was evaluated using the CaFRI framework, demonstrating moderate environmental considerations alongside key sustainability gaps. The method involved high energy consumption (>1.5 kW) and likely relied on energy-intensive equipment such as fume hoods and air conditioning, with no available data on carbon footprint measurement or emission factors. Samples required storage at -80 °C and short-distance transport (<10 miles) within Liverpool. Waste generation was minimal (<10 mL per sample); however, no recycling of reagents or solvents was reported. The method was semiautomated, required 2–3 personnel, and involved moderate use of hazardous solvents, indicating opportunities for greener optimization.


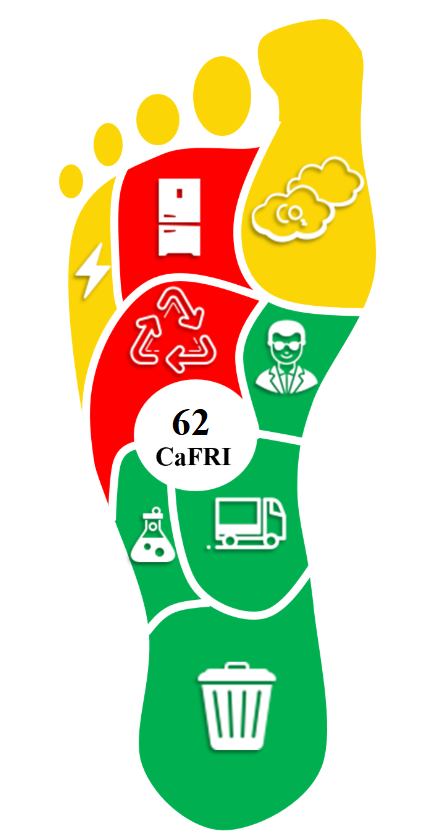


**Figure S1**: CaFRI assessment of the determination of favipiravir in human plasma by LC-MS/MS.

**Table S1:** Comparison between CaFRI and Labos 1point5.

| **Feature** | **CaFRI** | **Labos 1point5** |
| --- | --- | --- |
| Scope | Individual analytical method | Entire lab or institution |
| Functional unit | One analysis of a specific analyte in matrix | Yearly operation of lab or department |
| Output | Qualitative score (precautionary effort) | Quantitative CO₂e emissions |
| Use case | Awareness and guidance for greener methods | Carbon reporting and management |

**References**

1. Challenger E, Penchala SD, Hale C, Fitzgerald R, Walker L, Reynolds H, et al. Development and validation of an LC-MS/MS method for quantification of favipiravir in human plasma. J Pharm Biomed Anal. 2023;233:115436.
